# *Acinetobacter baumannii* Resistance: A Real Challenge for Clinicians

**DOI:** 10.3390/antibiotics9040205

**Published:** 2020-04-23

**Authors:** Rosalino Vázquez-López, Sandra Georgina Solano-Gálvez, Juan José Juárez Vignon-Whaley, Jorge Andrés Abello Vaamonde, Luis Andrés Padró Alonzo, Andrés Rivera Reséndiz, Mauricio Muleiro Álvarez, Eunice Nabil Vega López, Giorgio Franyuti-Kelly, Diego Abelardo Álvarez-Hernández, Valentina Moncaleano Guzmán, Jorge Ernesto Juárez Bañuelos, José Marcos Felix, Juan Antonio González Barrios, Tomás Barrientos Fortes

**Affiliations:** 1 Departamento de Microbiología del Centro de Investigación en Ciencias de la Salud (CICSA), FCS, Universidad Anáhuac México Norte, Huixquilucan 52786, Mexico; juan.juarezvi@anahuac.mx (J.J.J.V.-W.); jorge.abellova@anahuac.mx (J.A.A.V.); luis.padro@anahuac.mx (L.A.P.A.); andres.riverare@anahuac.mx (A.R.R.); mauricio.muleiroal@anahuac.mx (M.M.Á.); diego.alvarez@anahuac.mx (D.A.Á.-H.); valentina.moncaleano@anahuac.mx (V.M.G.); jorge.juarez@anahuac.mx (J.E.J.B.); 2 Departamento de Microbiología y Parasitología, Facultad de Medicina, Universidad Nacional Autónoma de México, Ciudad de Mexico 04510, Mexico; sgsolano@comunidad.unam.mx; 3 Medical IMPACT, Infectious Diseases Department, Mexico City 53900, Mexico; eunicevega.mi@gmail.com (E.N.V.L.); giorgio.franyuti@gmail.com (G.F.-K.); 4 Coordinación Ciclos Clínicos Medicina, FCS, Universidad Anáhuac México Norte, Huixquilucan 52786, Mexico; jose.felix@anahuac.mx; 5 Laboratorio de Medicina Genómica, Hospital Regional “1º de Octubre”, ISSSTE, Av. Instituto Politécnico Nacional 1669, Lindavista, Gustavo A. Madero, Ciudad de Mexico 07300, Mexico; jantgonzalez@issste.gob.mx; 6 Dirección Sistema Universitario de Salud de la Universidad Anáhuac México (SUSA), Huixquilucan 52786, Mexico; tbarrien@anahuac.mx

**Keywords:** *Acinetobacter baumannii*, antibiotic resistance, septicemia

## Abstract

*Acinetobacter baumannii* (named in honor of the American bacteriologists Paul and Linda Baumann) is a Gram-negative, multidrug-resistant (MDR) pathogen that causes nosocomial infections, especially in intensive care units (ICUs) and immunocompromised patients with central venous catheters. *A. baumannii* has developed a broad spectrum of antimicrobial resistance, associated with a higher mortality rate among infected patients compared with other non-*baumannii* species. In terms of clinical impact, resistant strains are associated with increases in both in-hospital length of stay and mortality. *A. baumannii* can cause a variety of infections; most involve the respiratory tract, especially ventilator-associated pneumonia, but bacteremia and skin wound infections have also been reported, the latter of which has been prominently observed in the context of war-related trauma. Cases of meningitis associated with *A. baumannii* have been documented. The most common risk factor for the acquisition of MDR *A baumannii* is previous antibiotic use, following by mechanical ventilation, length of ICU/hospital stay, severity of illness, and use of medical devices. Current efforts focus on addressing all the antimicrobial resistance mechanisms described in *A. baumannii*, with the objective of identifying the most promising therapeutic scheme. Bacteriophage- and artilysin-based therapeutic approaches have been described as effective, but further research into their clinical use is required

## 1. Introduction

The history of the genus *Acinetobacter* dates back to the 20th century, when the Dutch microbiologist Beijernick described an organism called *Micrococcus calcoaceticus* that was isolated from the soil by means of a medium enriched with calcium acetate [1,2]. 

The current genus designation, *Acinetobacter*, refers to the Greek concept “*Akinetos*”, which means “not mobile” [2]. Brisou and Prévot introduced this concept during 1954, with the objective of differentiating mobile from non-mobile organisms, mainly those belonging to the *Achromobacter* genus [3]. Although *Acinetobacter* is still described as a non-mobile microorganism, most strains exhibit “spasmodic” motility [4]. The nutritional and biochemical properties of *Diplococcus mucosus*, *Micrococcus calcoaceticus*, *Alcaligenes Haemolysans*, *Mima polymorpha*, *Moraxella lwoffi*, *Herellea Vaginicola*, *Bacterium anitratum*, *Moraxella lwoffi* var. Glucidolytica, *Neisseria winogradskyi*, *Achromobacter anitratus*, and *Achromobacter mucosus*, among others, were analyzed; concluding that all of them belonged to a common genus. In this way, the concept of *Acinetobacter* was introduced, but the subsequent subclassification into different species based on the phenotypic characteristics was not established at this point [4]. These findings resulted in the official recognition of *Acinetobacter* genus by the Taxonomy of Moraxella and Allied Bacteria Subcommittee during 1971 [5]. Later, in 1974, the genus was included in Bergey’s Manual of Systematic Bacteriology, along with the description of a single species: *Acinetobacter calcoaceticus* [5].

The *Acinetobacter* genus morphology consists of Gram-negative coccobacilli widely distributed in nature. Microorganisms included in it are characterized by being strictly aerobic, non-fermenting, nonfastidious, not mobile, catalase-positive, and oxidase-negative bacteria, with a 39% to 47% content of guanine–cytosine (GC) in their DNA sequence [2]. Within the genus, there are more than 50 species, which are mostly nonpathogenic environmental organisms [6]. However, among infectious species, *A. baumannii* has been described as the most virulent one [7], followed by *A. calcoaceticus* and *A. lwoffii* [8]. The phylogenetic description of the genus continuous to develop. Touchon et al. used 950 core protein families of the genus to build its phylogenetic tree with significant statistical support. In it, *A. brisouii* and *A. nectaris* are positioned as the taxa branching deeper in the genus. After this split, two large groups of taxa emerge, the first including *A. baumannii*, *A. parvus*, and *A. baylyi*, while the second one includes *A. lwoffii*, *A. johnsonii*, and *A. guillouiae*, among others. [9] The metabolic and ecological diversity of the species within the genus could be related to its ancient history. Touchon et al. suggested that its last common ancestor was a close contemporary of the last common ancestor of Enterobacteriaceae, which in turn emerged over 500 million years ago [10]. 

The first identification assay for *Acinetobacter* species was based on their biochemical profile. The use of molecular methods, specifically DNA–DNA hybridization [11], has been used to establish the identity of at least 33 different genospecies of *Acinetobacter* [12]. Among these genospecies, *Acinetobacter calcoaceticus* (genospecies 1), *Acinetobacter baumannii* (genospecies 2), *Acinetobacter* genomic species 3 (genospecies 3), and *Acinetobacter* genospecies 13TU (*Acinetobacter* genomic species 13), share phenotypic characteristics that make them difficult to differentiate. As a result, the above-mentioned have been grouped in the *Acinetobacter baumannii-calcoaceticus* (ACB) complex. Identification limited to the ACB complex can be misleading, as the genospecies in it differ in their biological and pathological characteristics. This remark must be taken into account, as *A. baumannii* (genospecies 2) represents the greatest clinical significance of the complex, while, for example, *A. calcoaceticus* (genospecies 1) has been considered to be an environmental pathogen rarely implicated as a cause of severe disease [13]. 

ABC organisms are characterized by having intrinsic antimicrobial resistance mechanisms that can be expressed constitutively or in response to antibiotic pressure. An analysis of susceptibility data for ABC organisms between 1995 and 2004 confirmed an increase in the percentage of resistance to all antimicrobial agents in four major classes of antimicrobials: fluoroquinolones (50–73% nonsusceptible), aminoglycosides (19–31% nonsusceptible), β-lactams (39–66% nonsusceptible), and carbapenems (9–39% nonsusceptible) [14]. Carbapenem resistance increase is of important concern, as this type of antimicrobial is often considered the last line of defense in treating resistant ABC. Of greater concern is the fact that a higher degree of bacterial resistance to antibiotics causes multiple classes of antimicrobial agents to become ineffective, this phenomenon is observed in bacteria called “multi-drug resistance” or “MDR” [15]. When reviewing the analysis of susceptibility data for ABC mentioned above, the authors considered MDR isolates as those with diminished susceptibility to all tested agents within three of the four major classes of antibiotic agents. They confirmed a significant increase in MDR ABC isolates (14% in 1995 to 26% in 2004) [14,15]. 

The most common MDR pathogens have been grouped in the acronym “ESKAPE” which stands for *Enterococcus faecium*, *Staphylococcus aureus*, *Klebsiella pneumoniae*, *Acinetobacter baumannii*, *Pseudomona aeruginosa*, and *Enterobacter cloacae*. The World Health Organization (WHO) declared that *A. baumannii* is one of the most serious of the ESKAPE organisms that effectively escape the effects of antibacterial drugs [16]. 

During the 1970s, clinical isolates of *A. baumannii* were susceptible to commonly used antibiotics, such as ampicillin, gentamicin, chloramphenicol, and nalidixic acid. However, in the late 1970s, it emerged as a significant nosocomial pathogen, mainly associated with the use of broad-spectrum antibiotics in hospitals [12]. Today, it shows resistance for most first-line antibiotics. Colistin and tigecycline remain the only antibiotics active against it and have become the last resort of treatment for multidrug-resistant (MDR) *A. baumannii*; however, colistin-resistant strains have been reported in different regions from around the world [17].

The increasing rates of antibiotic resistance can be attributed to multiple causes. There is a direct relationship between the emergence of resistant strains and the magnitude of antibiotic consumption [18]. Resistance mechanisms can be transmitted from one bacterium to another either longitudinally, when inherited from relatives, or horizontally, by means of plasmids. The latter may result in the transference of resistance among different species [19]. Inappropriate prescribing is also a determining phenomenon; studies have described that 30% to 50% of antimicrobial therapies are incorrectly prescribed [20,21]. Massive agricultural use of antibiotics, especially as growth supplements in livestock, is also a major concern; farm animals transfer antibiotics and resistant bacteria to humans through the intake of their meat, increasing the rate of antibiotic-resistant strains causing infections in humans. Another cause is the slow development of new antibiotics; the availability and low cost of antimicrobials has led to a considerable reduction of investment into new alternatives by the pharmaceutical industry [22]. 

Li-Kuang and collaborators analyzed clinical isolates of *A. baumannii*. 73.6% of such isolates were found to be resistant to quinolones (ciprofloxacin and levofloxacin), 71.3% to sulfonamides, and more than half (50–70%) to cephalosporins (cefazidime and cefepime), b-lactam/beta-lactamase inhibitor combinations (tazobactam–piperacillin), and carbapenems (doripenem, imipenem, and meropenem). It should be noted that just 26.7% demonstrated resistance against the glycycline antibiotic tigecycline. No resistance to colistin was observed [23]. 

There are numerous mechanisms through which antibiotic resistance develops. According to Gordon and Warehan, the most documented include alteration in the target sites, failure in the degradation-specific enzymes, perfusion defects and modification in multidrug effusion pumps, mainly associated with, among others [24]. 

With the objective of limiting the consequences of MDR microorganisms and developing better therapeutic approaches, the WHO in its report “Global Strategy for Containment of Antimicrobial Resistance” described two pillars of action. The first emphasizes preventing the dissemination of bacterial resistance, and the second, avoiding the accelerated emergence of new forms of resistance [25]. 

Early identification of patients exposed to risk factors favors the effectiveness of therapeutic outcome. In the particular case of nosocomial infections associated with imipenem-resistant *A. baumannii* strains, Lee and collaborators demonstrated the following as significant risk factors: previous intensive care unit (ICU) stay (OR, 21.54; 95% CI, 10.73 to 43.23) and prior exposure to third-generation cephalosporins (OR, 2.11; 95% CI, 1.13 to 3.95) or imipenem (OR, 9.18; 95% CI, 3.99 to 21.13), increased time at risk or days spent from admission to positive–culture date for *A. baumannii*-positive patients (OR, 1.02; 95% CI, 1.002 to 1.03), and age (OR, 1.03; 95% CI, 1.01 to 1.05) [26]. Lemos and collaborators described male sex as a significant risk factor for MRAB-C bacteremia (*p* < 0.001; OR 3.39, 95% CI (1.76–6.54)) [27].

Other studies have introduced advanced age, mechanical ventilation, renal failure, and prolonged hospital stay as important risk factors for the development of nosocomial *A. baumannii* infection [28,29]. Regarding risk factors for mortality, inappropriate empirical antibiotic therapy, high APACHE II score, severity of underlying disease, time of stay in the ICU, presence of fever and/or hypotension at the time blood sample for culture was obtained, and mechanical ventilation have been reported as significant. It must be noted that a high mortality rate has been associated with infections caused by carbapenem-resistant *A. baumannii* strains [30,31,32,33]. 

Once thought to be benign, *A. baumannii* is now considered a healthcare threat, mainly because of its potential to acquire multidrug, extensive drug, and even pandrug resistance phenotypes at previously unforeseen rates [34,35,36]. 

Infections caused by *A. baumannii* account for ~2% of all healthcare-associated infections in the United States and Europe; [37] however, these rates are twice as high in Asia and the Middle East [38]. Although infection rates are lower than those caused by other Gram-negative pathogens [34], ~45% of all isolates are considered to be MDR, a rate up to four times higher when compared to other Gram-negative pathogens, such as *P. aeruginosa* and *K. pneumoniae*. In particular, MDR rates reach about 70% in Latin America and the Middle East [35]. As a consequence to these concerning statistics, the WHO has included carbapenem-resistant *A. baumannii* in the “critical group” of all bacteria that represent greatest threat to human health, prioritizing research and development efforts for new antimicrobial treatments [39]. 

## 2. Pathogenicity Mechanisms

All the precise mechanisms involved in the establishment and progression of A. baumannii infection are unclear; however, some have been described.

Genomic studies comparing susceptible and resistant *A. baumannii* strains with the environmental strain, *Acinetobacter baylyi*, have identified genes involved in pilus biogenesis, iron uptake and metabolism, quorum sensing, and a type IV secretion system as making up part of the organism’s “virulome” [40]. 

The glicosiltranspherase (LpsB), one of the pathogenicity mechanisms identified in *A. baumannii*, is implicated in lipopolysaccharide (LPS) biosynthesis [41], a component of Gram-negative bacteria that is released after lysis or multiplication [42]. However, *A. baumannii* synthesizes lipooligosaccharide (LOS), which includes only core oligosaccharide and lipid A, rather than LPS, which would have an additional region of O-polysaccharide [43,44]. 

In most Gram-negative bacteria, lipid A is hexa-acylated. In contrast, *A. baumannii* lipid A is predominantly acylated hepta [44]; such hepta-acylation confers to *A. baumannii* resistance to cationic antimicrobial peptides (CAMPs) in vertebrate mucosal secretions and consequently allows desiccation survival, this being an important virulence factor of A. baumannii [44]. Outer membrane protein PagP is responsible for transferring the extra palmitoyl group to lipid A on Gram-negative bacteria, and particularly does so upon exposure to stressful factors [45]. However, *A. baumannii* lacks a gene that encodes a PagP homolog, so the synthesis of hepta-acylated lipid A molecules occurs independently of the PagP mechanism [44]. 

Lipid A confers a potentially toxic function and induces the pro-inflammatory cytokine expression in human monocytes mediated by the CD14 receptor, as well as TLR-2 and TLR-4 [46,47,48]. According to Erridge et al., the extent to which TLR-2 contamination with surface lipoproteins contributes to in vitro stimulation is debatable [47,48]. 

Additionally, *A. baumannii* expresses the phospholipase D, important for its development in human serum, and phospholipase C, which increases its cytotoxicity to epithelial cells [49,50]. 

*A. baumannii* is also characterized by a capsular polysaccharide, which decreases its adhesion to hydrocarbons, increases adhesion to epithelial cells, and facilitates protection against phagocytosis. The capsule consists of L-rhamnose, D-glucose, D-glucuronic acid, and D-mannose, polymerized and assembled by the genes *ptk* and *epsA* [51]. 

Outer membrane proteins (Omp) are also virulence key factors of *A. baumannii*, particularly OmpA, which is a major component of outer membrane vesicles [52,53]. This protein is directed towards an epithelial cell, adheres to it, and, after being taken in and reaching the nucleus, induces apoptosis through a process dependent on caspase-3 and mediated by the activation of upstream initiators such as caspase-8 and caspase-9 [54,55,56]. Purified OmpA provokes a Th_1_-mediated immune response [57] and upregulates inducible nitric oxide synthase (iNOS) via a Toll-like receptor (TLR)-2-mediated pathway [58]. 

Mitochondrial changes are also initiated, leading to the translocation of cytochrome C into the cytosol and the initiation of an apoptotic cascade [55]. Choi et al. have suggested that OmpA also degrades chromosomal DNA by DNAse I-like enzymatic activity [59]. OmpA has been also described as an important complement-regulator-acquiring surface protein, allowing *A. baumannii* to evade complement attack [60]. This outer membrane protein plays a partial role in the development of biofilms on plastic, but is required for attachment to fungal filaments and epithelial cells [61]. 

*Acinetobacter baumannii* readily adheres both to biological and abiotic surfaces, on which it is able to form biofilms [62,63,64]. The disruption of the *csuC* and *csuE* open reading frames (ORFs) has been linked to *A. baumannii* strains that lack pili and biofilm formation, suggesting that these genes are involved in both virulence factors [65]. Appendages involved in its synthesis are type IV pili, flagella, curli, and fimbriae [40]. There is evidence to suggest that biofilms increase *A. baumannii* survival time in dry environments (these organisms can live for an average of 20 days at a relative humidity of 31%) [66]. Desiccation resistance, which is the ability to maintain viability under dry conditions, varies among clinical isolates of *A. baumannii*, with some isolates remaining viable for almost 100 days. Such ability is multifactorial and not yet fully defined [34,67,68]. It must be noted that biofilm formation could be associated with increased resistance against immune host response [30]. 

For the maturation of such biofilm, a protein associated with biofilm (BAP) located in its external membrane is required; it provides greater thickness and volume, in this manner facilitating intercellular adhesion [69]. *A. baumannii* contains a locus that encodes proteins which synthesize cell-associated poly-β-(1-6)-*N*-acetylglucosamine (PNAG) polysaccharide; PNAG is a key component to securing the organism’s stability in stressful environments [70]. 

Initial adhesion depends on functional fimbriae and pili, followed by the production of exopolysaccharide, which suppresses the activity of neutrophils and contributes to serum resistance. Variation in the expression of factors involved in these and other pathways may account for the differing capacity of strains to colonize or infect the host environment [24]. Once adhered, *A. baumannii* invades and induces apoptosis of eukaryotic cells [55], a property attributed to the activity of OmpA (Omp36), which is trafficked to both the mitochondria and the nucleus [54,55]. 

Human multidrug-resistant *A. baumannii* strain is characterized, as many other Gram-negative bacteria, by having a N-acylhomoserine lactone (AHL)-mediated quorum-sensing regulatory mechanism, which is known to be involved in biofilm formation [71], host-cell gene expression modification [72] and non-ribosomal peptide synthase (NRPS) encoding, which in turn results in toxins, siderophores, antibiotics, and pigments [40]. 

Furthermore, bacterial pathogenicity is closely related to the capacity to use specific iron acquisition strategies [73], such as the production of aerobactines and iron-dependent external membrane proteins [74]. Siderophores/hemophores, high-affinity iron molecules released outside of cells that compete with host binding proteins for essential iron, have been identified in human multidrug-resistant *A. baumannii* strains [74]. These are essential for the survival and growth of the pathogen under low-iron-content circumstances [40,73]. 

## 3. Antibiotic Resistance Shown by *A. baumannii*

Different *A. baumannii* strains are endemic to different regions around the world [75]. MDR strains survive antimicrobials by means of various mechanisms, each of them specific against particular types of drugs.

## 4. Aminoglycosides

Aminoglycosides bind to the RNA 16S of the ribosomal 30S subunit. The most studied mechanism of resistance to this type of antibiotic in *A. baumannii* strains is the production of aminoglycoside modifier enzymes. Three different functional groups of modifier enzymes are known, which include aminoglycoside acetyltransferases (AAC), for example AAC (6′)-Ih (which also confers resistance to gentamicin and amikacin) [76,77], aminoglycoside phosphotransferases (APH), for example APH (3′)-IA (which confers resistance to gentamicin) [78], and aminoglycoside adenililtransferases (ANT), for example ANT (2′′)-IA [77]. The production of RNA 16S ribosomal methyltransferase, especially ArmA, the first of its type to be discovered in a clinical isolate, is a mechanism of resistance to aminoglycosides that appears to be emerging [79]. *A. baumannii* strains that produce ArmA are highly resistant to gentamicin, amikacin, and tobramycin [80,81]. 

On the other hand, a study conducted in Korea by Choy et al. demonstrated the presence of aminoglycoside-resistance genes in 61 out of 75 *Acinetobacter* isolates from two Korean hospitals. *A. baumannii* isolates carrying the genes encoding aminoglycoside-modifying enzymes ant(3″)-Ia, aac(6′)-Ib, aph(3′)-1a, aac(3)-Ia, and aph(3′)-VI, and 16S ribosomal RNA (rRNA) methylase ArmA were resistant to amikacin, gentamicin, isepamycin spectinomycin, streptomycin, and tobramycin [82]. 

## 5. Carbapenems

Carbapenems have the broadest spectrum among all β-lactams and are mainly used as a treatment in infections caused by Gram-negative bacteria [83]. Overexpression of the carbapenem-hydrolyzing oxacillinase (OXA)-51-like-B-lactammase [84,85] and ArmA RNA 16S ribosomal methyltransferase are among the mechanisms that confer resistance to carbapenems among *A. baumannii* strains [86]. 

Increasing emergence of carbapenem resistance, frequently mediated by production of Ambler’s class D β-lactams (OXA), in *A. baumannii* is a major concern [87,88]. Many of them are found as part of integrons [89,90]. *A. baumannii* can present intrinsic chromosomal OXA-51-like, and four additional groups of OXA acquired carbapenemases, including OXA-23, 24 (OXA-40-like), -58-like, and -123-like [91]. OXA-23 has been associated with greater dissemination and production of carbapenem resistance with clinical consequences [92]. It has been documented that OXA-24 has moderate hydrolytic activity against carbapenems [93]. 

*A. baumannii* produces the group of chromosomally encoded carbapenemases OXA-51 in a basal manner, so it is not a cause of resistance by itself. However, if the transposition of ISA*ba1* or ISA*ba9* occurs upstream of the gene of the group OXA-51, overexpression is initiated [94,95]. 

It should be noted that the OXA group of carbapenemases confer resistance to oxacillin and carbapenems, but not to cephalosporins. However, in most clinical isolates, the acquired carbapenem resistance was mediated by the presence of *blaOXA* genes, and resistance to cephalosporins was already present due to other *bla* type gene like *blaAmpC* [96]. Non-OXA carbapenemases have also been reported in *A. baumannii*, although they are more frequent in Enterobacteriaceae. For example, Ambler’s class B metallo-β-lactamases have been identified. In this classification, the NDM (New Delhi metallo-β-lactamase) group of enzymes includes IMP (imipenemases), VIM (Verona imipenemase), and SIM (Seoul imipenemase), which are types of metallo-β-lactamases not common in *A. baumannii* strains [97,98,99]. 

Fernández-Cuenca et al. suggested that the reduced expression of penicillin-binding protein (PBP) 2 is also an important mechanism involved in the acquisition of carbapenem resistance [100]. 

## 6. Fluoroquinolones

*A. baumannii*’s fluoroquinolone resistance mechanism consists of substitutions in the quinolone resistance-determining regions (QRDRs) of DNA gyrase and DNA topoisomerase IV, which interfere with the fluoroquinolones’ union to their target proteins [86]. Overexpression of efflux active pumps can also cause moderate resistance by itself and increase resistance in strains with RDRQ substitutions [101]. 

## 7. Cephalosporins

Many of the clinical isolates of *A. baumannii* are resistant to cephalosporins. A new Ambler’s class C β-lactamase was found in a clinical isolate recovered from a hospital in Cleveland OH, USA. This enzyme, also expressed in *Escherichia coli DH10B*, demonstrated greater resistance against ceftazidime and cefotaxime than cefepime. After a phylogenetic analysis of this new enzyme and other class C β-lactamases described in *A. baumannii*, *Acinetobacter pittii*, and *Oligella urethralis*, it was found to define a unique class of class C enzymes. In this manner, a uniform designation was proposed for these cephalosporinases: *Acinetobacter*-derived cephalosporinases (ADC). The new enzyme was named ACD-7 β-lactamase, as six related cephalosporinases had been described before [102]. Unlike most chromosomally produced class C β-lactamases, *A. baumannii* AmpC ADC-7 cannot be induced by cefoxitin [102]. According to Lopes et al., the ADC expression can be increased if there is an insertion of the ISA*ba1* or ISA*ba125* sequence upstream of the ADC gene, so there is a higher promoter activity compared to the activity of the native promoter [103,104]. As a result, an elevation of the minimum inhibitor concentrations of the different cephalosporins is required, except cefepime, which is not a substrate of the β-lactamase class C, including ADC. It has been reported that there are strains of *A. baumannii* that produce extended-spectrum class C β-lactamases such as ADC-33, which do confer resistance against cefepime, as well as against other cephalosporins [105]. 

## 8. Sulbactam

PBP2 (penicillin-binding protein) has been shown to have the highest affinity for penicillin and beta lactamase inhibitors. Sulbactam binds to *A. baumannii*’s PBP2 to initiate an effect against the microorganism [106]. The resistance of *A. baumannii* to sulbactam is associated with a reduced expression of PBP2 [100]. Additionally, it has been demonstrated that the role of β-lactamase synthesis encoded in the gene BlaTEM-1, contributes to sulbactam resistance in *A. baumannii* [107]. 

It is important to take into account that the potential therapeutic usefulness of sulbactam has been reported as superior compared to other β-lactamase inhibitors, such as clavulanic acid and tazobactam. β-lactamase inhibitors have intrinsic activity but are not capable of enhancing the activity of β-lactams against *A. baumannii* [108]. 

## 9. Rifampicin

Rifampicin binds to the active site of bacterial RNA polymerase, inhibiting the transcription process. The mechanism of evasion to this medicine is the substitution of amino acids in the β-subunit of this target protein. Elevated minimum inhibitory concentrations in *A. baumannii* have been associated with *rpoB* mutations [109]. In addition, active efflux and enzymatic modification by the ADP ribosyltransferase of rifampicin (ARR-2) contribute to resistance against rifampin in *A. baumannii* strains [109,110]. 

## 10. Tetracyclines

Resistance against tetracyclines is mediated by various mechanisms, including active efflux of the antimicrobial mediated by resistance proteins in the bacterial cytoplasmic membrane and inhibition of ribosomal and tetracycline binding [111]. 

Tigecycline, which was designed to prevent most resistance mechanisms, is prone to effusion generated by the resistance-nodulation-division (RND)-type efflux pumps produced by most clinical isolates of *A. baumannii* [112]. Hornsey et al. described high MICs of tigecycline being correlated with the elevated expression of *adeABC*. Clinical isolates resistant to tigecycline, when having their *adeB* interrupted, once again showed full susceptibility to this antibiotic [113]. 

## 11. Polymyxins

As antibiotic resistance increases among Gram-negative bacteria, especially *P. aeruginosa*, *A. baumannii*, and *K. pneumoniae*, effective antimicrobial therapeutic approaches have been limited. Now, the effectiveness of the clinical application of colistin, a polymyxin that was discovered more than 50 years ago, is being re-evaluated [114]. According to the CDC (Centers for Disease Control and Prevention of the U.S. Department of Health and Human Services), because polymyxins are generic drugs, there is limited updated data on their proper dosing [115]. 

Colistin (Polymyxin-E) interacts with lipopolysaccharide’s (LPS) lipid A. Its modification results in acquired polymyxin resistance. The most commonly reported modification method is the addition of a phosphoethanolamine residue to the hepta-acylated form of lipid A, removing negative charges and lowering the affinity LPS for polymyxins [116,117]. Another mechanism through which *A. baumannii* develops such resistance is by complete loss of the initial LPS [118]. According to Biswas et al., the combination therapy of polymyxin–rifampicin is being studied for the treatment of MDR Gram-negative bacteria. In most studies, the colistin–rifampicin combination has shown a 100% synergy when facing MDR *A. baumannii* [119]. 

## 12. Resistance Genes

The various identified resistance genes in *A. baumannii* can be constitutive or acquired by means of integrons, transposons, and plasmids. They encode both the enzymes that modify the antibiotic molecules and the modifications of the antibiotic target sites. These genes also code for the efflux pump proteins and porin channels of the cell membrane, both systems related to the decrease in the intracytoplasmic concentration of the antibiotic. [120]. The *AdeB* gene controls the expression of AdeB proteins, which are members of the resistance-nodulation-division (RND) efflux pump superfamily, thus regulating the bacterial internal drug efflux pump that plays a significant role in drug resistance [121,122]. Another study revealed that mutations in the genes of lipid A biosynthesis (LpxA, LpxC, and LpxD) may lead to significant increase in the resistance to colistin. Additionally, extended-spectrum β-lactamase genes blaTEM, blaSHV, and blaCTX-M have been described among *A. baumannii* [123]. Most clinical isolates (98%) analyzed by Benmahmod et al. carried blaOXA-23-like and blaOXA-51-like simultaneously. In such isolates, coexistence of carbapenem-hydrolyzing β-lactamases also was detected (blaKPC, bla GES, blaNDM, blaSIM, blaVIM, blaIMP) [124]. 

## 13. Treatment

The World Health Organization (WHO) has considers antibiotic multiresistance to be one of the three greatest health threats of the century [125]. 

Treatment against *A. baumannii* infections is limited by the recurrent antimicrobial resistance rate. In particular, the ACB complex (*A. calcoaceticus*, genospecies 1; *A. baumannii*, genospecies 2; *A.* genomic species 3, genospecies 3; *A.* genospecies 13TU, genospecies 13) is responsible for approximately 80% of infections and has shown resistance to all Gram-negative antimicrobial agents [126]. The antimicrobial resistance that characterizes *A. baumannii*, is related to its capacity to react rapidly to challenges issued by antimicrobials. Thus, the widespread use of antibiotic therapies in healthcare facilities, especially extended-spectrum cephalosporins and quinolones, represents an important factor to be considered [127]. In vitro susceptibility tests have become a useful tool for determining the adequate antibiotic treatment [128]. *A.-baumannii*-associated nosocomial infections cause high mortality; thus, decreasing this mortality is a key therapeutic objective. In order to successfully overcome antimicrobial resistance, a wide variety of therapeutic combinations have been considered as first-line treatments (Table 1) [129].

While therapeutic possibilities against *A. baumannii* decrease, patient mortality and in-hospital stays increase, particularly due to pneumonia and inadequate antimicrobial treatment. A particular antimicrobial therapeutic scheme against *A. baumannii* bacteremia has not been established: there is a lack of adequate studies. Nevertheless, the usual choice is an active β-lactam alone or in combination with an aminoglycoside. Treatment involving imipenem has also been reported to be useful [126,129]. 

Other useful therapeutic schemes include colistin/imipenem, colistin/meropenem, colistin/rifampicin, colistin/tigecycline, colistin/sulbactam, colistin/teicoplanin, and imipenem/sulbactam [126,149,150].

As reported by Murray et al., the agents with the most antimicrobial activity are imipenem/cilastatin, amikacin, ampicillin/sulbactam, colistin, and tetracyclines, but no one agent appears superior to any other [126]. Antimicrobial combination therapies against *A. baumannii*, including colistin/rifampicin [139,143], colistin/minocycline [139], colistin/carbapenem [136,139,140], colistin/sulbactam [136], colistin/tigecycline [141,142,151,152], colistin/daptomycin [144], colistin/fusidic acid acid [145,153], and colistin/teicoplanin [144,146], are synergistic in vivo or in vitro against such bacteria [149]. 

In order to prevent bacterial resistance against these drug combinations, the antimicrobials should not be administered in an inhaled form [154]. 

### 13.1. Carbapenem (Imipenem and Meropenem) and β-Lactam Inhibitors (Sulbactam)

Common antimicrobial treatments against *A. baumannii* infections include carbapenems; however, the prevalence of isolates resistant to carbapenems (carbapenem-resistant *A. baumannii* (CRAB)) is increasing, leading to longer in-hospital stays and a higher mortality rate [155]. 

Imipenem’s mechanism against Gram-negative bacilli relies on its capacity to penetrate through the outer cell envelope and to bind with high affinity to certain penicillin-binding protein (PBP) targets. Imipenem and meropenem have high affinity to PBP1a, PBP1b, PBP2, PBP4, and PBP5 [156]. The resistance to a wide variety of β-lactamases, a direct consequence of its 6 a-hydroxyethyl side chain and the resistance to hydrolysis by the newer enzymes expressed by TEM-2 and SHV-1, are additional mechanisms responsible for the broad-spectrum activity of imipenem [157,158]. 

There are three β-lactamase inhibitors commonly used in the CRAB infections for their intrinsic activity against *A. baumannii*: sulbactam, tazobactam, and clavulanic acid [108]. Sulbactam, as studies have reported, is the most effective and may represent an alternative treatment option for infections caused by MDR *A. baumannii* strains [2,108,159]. 

Several studies have demonstrated sulbactam/ampicillin to be a highly effective combination against pneumonia, blood, and nosocomial infections caused by *A. baumannii* [160,161]. In vitro studies have shown that sulbactam enhanced its therapeutic activity when combined with cefepime, imipenem, meropenem, amikacin, rifampin, and ticarcillin–clavulanate [137,138]. Temocin et al. mentioned, in addition to the previously listed combinations, the synergistic effect of sulbactam with ampicillin and cefoperazone. Further research into the combination with tigecycline is recommended [137]. 

### 13.2. Tetracyclines (Minocycline and Doxycycline) and Glycylcyclines (Tigecycline)

Tetracycline therapy is a good option when facing carbapenem- and sulbactam-resistant *A. baumannii* strains [130]. When treating pneumonia, doxycycline/amikacin combination is considered viable; however, it has been suggested it does not improve the results obtained by imipenem monotherapy [162]. 

Both doxycycline and minocycline are effective against ventilator-associated pneumonia (VAP) caused by MDR *A. baumannii* strains [130]. It must be noted that ACB complex strains from a U.S. Military Hospital were shown to be more susceptible to minocycline that to other tetracyclines and tigecycline, as documented by Akers et al. [131]. Although Holloway et al. suggest further investigation on doxycycline for the treatment of MDR *A. baumannii* infections [163], Griffith et al. support the effective use of minocycline in treating MDR *A. baumannii* infections. One of minocycline’s benefits, in contrast to imipenem, colistin, and tigecycline, is that it can be given orally to the patient [132]. 

Tigecycline is a broad-spectrum drug and was the first drug in the glycylcycline antibiotic class, approved in 2005 by the United States Food and Drug Administration (US-FDA) for clinical use [134]. Although it has high structural similarity to minocycline, molecular changes in its structure guarantee less susceptibility to generating resistance when compared with other tetracycline antibiotics [134]. Tigecycline binds to the 30S ribosome subunit and blocks the transfer RNA input to avoid protein synthesis, limiting bacterial growth. Its activity is effective against Gram-positive and Gram-negative bacteria, and has shown good antimicrobial activity against MDR *A. baumannii* strains, including those resistant to imipenem, associated with ICU nosocomial infections. Gram-positive bacteria, such as *S. aureus*, *Enterococcus spp*, coagulase-negative *Staphylococcus*, and *P. aeruginosa* have shown greater susceptibility to tigecycline than Gram-negative bacteria [134,164,165,166]. 

Nevertheless, Hakyemez et al. analyzed 56 *A. baumannii* strains, 59% from ICU (most commonly isolated in reanimation ICU) and did not identify any *A. baumannii* strain resistant to tigecycline [135]. 

### 13.3. Polymyxins (Colistin and Polymyxin B) and Therapeutic Combinations (Rifampicin and Teicoplanin)

Colistin (polymyxin E)-based therapy, which is often used in combination, has been described as a last resort for the treatment of MDR *A. baumannii*. It should be noted that colistin-resistant *A. baumannii* strains have been reported in various regions [167,168]. 

Polymyxin E (colistin) and polymyxin B are increasingly being used as the last-line therapeutic option for increasing infections against MDR Gram-negative bacteria such as *Pseudomonas aeruginosa*, *Acinetobacter baumannii*, and *Klebsiella pneumonia* [169]. These antimicrobials have various mechanisms through which they kill bacteria. First, they can induce membrane lysis. An electrostatic interaction is generated, allowing the insertion of a particular polymyxin molecule into the outer membrane of the bacteria. The packing of the adjacent lipid A is weakened, facilitating the formation of destabilized areas through which polymyxin crosses the outer membrane. Finally, the integrity of phospholipid bilayer of the inner membrane is compromised, resulting in lysis and cell death. The second mechanism is a vesicle–vesicle contact pathway, through which polymyxin induces a lipid exchange between the outer and the inner membranes, potentially causing an osmotic imbalance and leading to cell death. In the third and last mechanism, polymyxins induce rapid killing of Gram-negative bacteria; these antimicrobials induce hydroxyl radical production, which accumulate excessively and lead to bacterial cell death through oxidative damage to DNA, lipids, and proteins [170,171,172]. 

It must be noted that colistin is parenterally administered for its inactive prodrug, while polymyxin B is available for direct parenteral administration, thus providing a quicker effect [173]. 

Colistin is used against MDR Gram-negative bacteria, particularly *A. baumannii*, in combination with other drugs, such as colistin–rifampin, colistin–meropenem, colistin–minocycline, colistin–carbapenem, colistin–sulbactam, and colistin–teicoplanin [139,174,175]. Evidence shows that *Acinetobacter* isolates were previously susceptible to colistin, however, now they are characterized by resistance and, in some cases, dependence on this antimicrobial. Patients suffering from *A. baumannii* infections dependent to colistin are associated with a high rate of failure in treatment [176,177]. 

Dubrovskaya et al. suggest further research regarding the nephrotoxic effect associated with polymyxin B. Taking this into account, healthcare providers may limit the toxic effects of therapeutic combination in a patient with a MDR infection [178]. 

However, in vitro studies have demonstrated the effective use of polymyxin B in combination with certain antibiotics to treat multidrug-resistant infections caused by *A. baumannii*. It has been successful when used in combination with carbapenems (doripenem, meropenem or imipenem), rifampicin, and tigecycline [179,180,181]. As tigecycline also has microbiological activity against multidrug-resistant isolates, its combination with polymyxin B against *A. baumannii* infections is common [179]. 

### 13.4. Trimethoprim (TMP)–Sulfamethoxazole (SMX)

In vitro studies have demonstrated the efficacy of TMP-SMX combination against CRAB infections [133]. Reducing the production of tetrahydrofolate (FAH4), essential to cellular growth and survival, by inhibiting the activity of reductases that under normal circumstances maintain FAH4 pools, is the characteristic mechanism by which TMP kills bacteria. Its antimicrobial effect is enhanced when used in combination with SMX, as it simultaneously blocks the synthesis of FAH2 [182,183]. 

### 13.5. Bacteriophages, Endolysin (Artilysin)

Bacteriophages are bacteria-infecting viruses with precise bacteriolytic activity. MDR isolates of *A. baumannii*, including CRAB, can be targets for such bacteriophages, so they are being considered as a useful alternative to face drug resistance. No deleterious side effects associated with their use have been reported [147,148]. Phage Bφ-C62 has shown the strongest lytic activity [147]. It must be noted that Schooley et al. reported the reversal of a diabetic patient’s downward clinical trajectory caused by necrotizing pancreatitis complicated by MDR *A. baumannii* infection after the administration of these bacteriophages intravenously [184]. However, this therapy’s high cost and low experimental evidence limit its usage.

Endolysins are soluble enzymes that are produced and released by double-stranded DNA phages at the end of their replication cycle to degrade the peptidoglycan of the bacterial host, thus leading to cell lysis and the release of progeny virions. A second lysis factor is involved in this process, known as holing [185]. Its fast action, high specificity, efficacy, low toxicity, and low probability to produce resistance make these endolysins a potential efficient treatment for MDR bacterial infections [186]. The mechanism involved is similar to that of a peptoglycan hydrolase. The endolysins are more effective against Gram-positive bacteria. On the other hand, Gram-negative bacteria are naturally protected from endolysins by their outer membrane, which protects the target of endolysins: the peptoglycan. In order for endolysins to be effective against Gram-negative bacteria, they have to be genetically modified into “artilysins”. Huang et al. expect that phage-encoded endolysins could become very useful antibacterial agents against pathogens. Specifically, endolysin gene plyB1, which codes for endolysin from the Abp1 phage, has potential as an antibiotic against MDR *A. baumannii* [187]. 

## 14. Conclusions

*A. baumannii* has developed multiple antibiotic resistance mechanisms, increasing the harmful consequences of its pathogenic potential and representing an important challenge for patients and healthcare providers. Clinicians have to take into account all documented risk factors and the experimental synergistic activity of different antimicrobials in order to achieve a more successful treatment for patients with MDR *A. baumannii* infections. Effectiveness of treatment relies mostly on accurate antimicrobial combinations with sulbactam or polymyxin E (colistin). On the other hand, monotherapy has been described as successful against particular *A. baumannii* strains in certain clinical scenarios. Although the use of β-lactam alone or in combination with an aminoglycoside is common, increasing rates of resistant strains demand the use of an effective β-lactamase inhibitor, such as sulbactam. Nevertheless, sulbactam-resistant strains have been reported. Additionally, strains resistant and dependent to polymyxin use could exceed its efficacy in antimicrobial combinations. Therefore, the use of tigecycline, alone or in combination, or Phage Bφ-C62 remain as the antimicrobials with most potential against MDR *A. baumannii* infections. The WHO included CRAB (carbapenem-resistant *A. baumannii*) in the “critical group” of bacteria that represent the greatest threat to human health and recommended further research to face its clinical impact; however, in addition to investment in new therapeutic alternatives, efforts must be oriented towards limiting the indiscriminate hospital use of broad-spectrum antibiotics. Antimicrobial resistance will become a major concern in years to come, and its expected clinical impact will only be limited if multidisciplinary teams gather around the world to develop innovative solutions not only at a molecular level, but also at an institutional one.

## Figures and Tables

**Table 1:** Effective Antibiotic Therapies against *A***.** *Baumannii* according to Therapeutic Groups and Isolated Strains in Specific Clinical Scenarios.

		Effective Antibiotic Therapy
Isolated strains in specific clinical scenarios	Carbapenem- and sulbactam-resistant strains.	Doxicycline or minocycline, which in turn is more effective [130,131,132]
	Carbapenem-resistant strains.	TMP-SMX [133]
	MDR strains in the ICU.	Tigecycline [134,135].
Therapeutic groups	Synergistic therapeutic combinations with β-lactamase inhibitor sulbactam.	Sulbactam/cefepime, sulbactam/meropenem, sulbactam/amikacin, sulbactam/rifampin, sulbactam/ticarcillin–clavulanate, sulbactam/ampicillin, sulbactam/colistin [136], and sulbactam/cefoperazone [137,138].
	Synergistic therapeutic combinations with polymyxin E (colistin).	Colistin/carbapenem [136,139,140] colistin/minocycline [139], colistin/tigecycline [141,142], colistin/rifampin [119,139,143], colistin/sulbactam [136], colistin/daptomycin [144], colistin/fusidic acid [139,145], and colistin/teicoplanin [144,146].
	Last-line therapeutic scheme.	Polymyxin E (colistin) in combination with rifampin or polymyxin B with tigecycline [119].
	Alternative against increasing antibiotic resistance.	Phage Bφ-C62 [147,148].
